# Risk factors and prevalence of hypertension in older adults from south-eastern Poland: an observational study

**DOI:** 10.1038/s41598-024-52009-3

**Published:** 2024-01-16

**Authors:** Justyna Leszczak, Ewelina Czenczek-Lewandowska, Muhammad Asif, Joanna Baran, Artur Mazur, Justyna Wyszyńska

**Affiliations:** 1 Institute of Health Sciences, Medical College, University of Rzeszów, Rzeszów, Poland; 2 Department of Statistics, Govt. Associate College, QadirPurRaan, Multan, Pakistan; 3 Institute of Medical Sciences, Medical College, University of Rzeszów, 35-959 Rzeszów, Poland

**Keywords:** Health care, Risk factors

## Abstract

The aim of this study was to assess the prevalence of hypertension and to investigate risk factors linked to hypertension in older adults. An observational study was conducted in a group of adults between 60 and 85 years of age, living in south-eastern Poland. In line with the specific inclusion criteria, 80 women and 29 men were enrolled for the study (109 adults). Participants’ body weight, height, and body fat percentage (BFP) were assessed using a bioelectrical impedance analysis, blood pressure was measured using automated oscillometric sphygmomanometer, moderate-to-vigorous physical activity (MVPA) and sedentary time were assessed using a tri-axial accelerometer, whereas data related to socio-economic and lifestyle factors were collected using a self-report technique. Arterial hypertension was found at a rate of 16% in participants with normal body weight, 22% in those with overweight and 85% in those with obesity. Body mass index (BMI) and BFP correlated significantly with systolic blood pressure (SBP) and diastolic blood pressure (DBP). The highest median SBP and DBP values were found in the group of participants with obesity, and the lowest values were identified in those with normal body weight. Out of all the investigated socio-economic risk factors linked to hypertension, education level was the only one that showed significant associations. A logistic regression analysis was performed to check which factors were most strongly associated with hypertension in the study group. The stepwise method showed that hypertension was more common in participants with a higher BMI, and BFP and in those who did not meet MVPA recommendation.

## Introduction

Hypertension is one of the main modifiable risk factors linked to cardiovascular diseases, and its prevalence as well as severity both increase with age^1^. US National Health and Nutrition Examination Survey (NHANES) showed that 70% of older adults over 65 years of age have hypertension^2^. The global prevalence of hypertension in people aged 30–79 years was estimated at a level of 1.27 billion in 2019, which corresponds to 32% women and 34% men globally^1^. This number will continue to grow with the aging of our population. This high percentage of hypertension worldwide is not related to the income status, i.e. it occurs in lower, middle and higher income countries^3^. The prevalence of hypertension increases with age; in the older adults over 60 years of age it exceeds 60%^3^. With age the levels of physical activity decline, corresponding to generally quieter lifestyles and increasing body weight, consequently the incidence of hypertension in the rapidly aging society will continue to increase worldwide. It is estimated that by 2025 the percentage of people with hypertension will increase by 15–20%, reaching the number of nearly 1.5 billion^4^.

In 2018, the European Society of Cardiology (ESC) and the European Society of Hypertension (ESH) published guidelines for the management of arterial hypertension in adults. One of the goals of these guidelines was to provide pragmatic recommendations to improve the detection and treatment of hypertension^5^. This document highlighted the challenges related to managing blood pressure in older patients, i.e., those aged 60 + years as defined by the American College of Physicians and American Academy of Family Physicians. Based on the ESC/ESH recommendations, we conducted a study focusing on older adults in Poland. The purpose of this study was to assess the prevalence and risk factors for hypertension in older adults from south–eastern Poland. Understanding the risk factors for hypertension is crucial for developing effective intervention programs for the prevention of this disease, as evidence has shown that by controlling blood pressure it is possible to substantially decrease a risk of premature morbidity and mortality^6^.

## Materials and methods

### Study design and population

An observational study was conducted in a group of older adults attending the University of the Third Age. A non-random sampling method was used. All participants who met the inclusion criteria were recruited. The age of participants ranged between 60 and 85 years. Voluntary informed consent was obtained in writing from participants prior to the study. The protocol was approved by the Bioethics Committee at the Medical Collage of the University of Rzeszów, Poland (ref. no. 2/02/2019), and the study was conducted in accordance with ethical standards laid down in an appropriate version of the Declaration of Helsinki.

Based on data published by the Polish Central Statistical Office, in 2019 there were approximately 40,000 older adults (over 60 years of age) in Rzeszów, Poland. Assuming a confidence interval of 95%, population proportion of 60% as well as a 10% margin of error, it was determined the required sample should include at least 92 participants. At 95% confidence interval, fraction size of 0.6, population size of 40,000, sample size of 109 participants, the maximum error is 10%, which means the power of the test is 1-Beta (10%), i.e., 0,9.

A total of 150 individuals were invited to enrol for the study. Following the recruitment process based on the inclusion and exclusion criteria, the study involved 82% (n = 123) of those invited to take part in the study. The attendance rate was higher in women than in men (80 women, 29 men). The following eligibility criteria were used for inclusion in the study: the individuals’ written consent to participate, age ≥ 60 years, health status allowing for the examinations to be carried out, as well as no metallic implants, prosthesis or pacemaker. The participants were excluded from the final analyses if they failed to participate in any of the measurements.

### Anthropometric measurements

Participants’ body weight and height were measured using a standard protocol and equipment which was calibrated before and during the period of data collection. Body height was measured in an upright position, barefoot, to the nearest 0.1 cm using a portable stadiometer (HR-200, Tanita). Body mass was assessed with an accuracy of 0.01 kg using a body composition analyser (780 MA, Tanita). Body mass index (BMI) was calculated as weight in kilograms divided by height in meters squared (kg/m^2^). Based on the BMI values, the participants were divided into subgroups: normal weight (BMI: 18.5–24.9), overweight (BMI:25.0–29.9) and obesity (BMI > 30.0)^7^.

### Blood pressure

Blood pressure (BP) was examined according to the current protocol guidelines for BP measurement after the participant had rested for 10 min^8^. Three readings were taken at intervals of one or two minutes, using an automated oscillometric sphygmomanometer from Welch Allyn Inc., 4200B-E2 (Skaneateles Falls, NY, USA) equipped with a set of cuffs of various width. The cuff was placed on the participant’s arm and positioned at the heart level; the measurements were performed in the same conditions with the participant seated with his/her back against the chair and forearm supported on the table. Blood pressure classifications were defined in line with the 2018 ESC/ESH Guidelines for the management of arterial hypertension in adults. It was assumed hypertension corresponded to systolic blood pressure (SBP) ≥ 140 mm Hg and diastolic blood pressure (DBP) ≥ 90 mm Hg. The acquired measures were classified in four subgroups corresponding to ideal blood pressure (< 120/80 mm Hg), normal (120–129/80–84 mm Hg), high normal (130–139/85–89 mm Hg) and hypertension (> 140/90 mm Hg)^9^.

### Body fat percentage

Body fat percentage (BFP) was assessed using a bioelectrical impedance analysis (BIA) device (780 MA, Tanita). The BIA is a reliable and accurate tool for the measurement of body fat in the adult population^10^. Participants were informed about the need to refrain from food and drink for at least 8 h before the examination, since food or beverage consumption may decrease impedance by 4–15 Ω over a 2–4 h period after meals, representing an error smaller than 3%^11^.

Based on their BFP, the subjects were divided into two subgroups, corresponding to ‘no excess adiposity’ and ‘excess adiposity’. The cut-offs for BFP were defined as 25.8% and 37.1% for men and women, respectively^12^.

### Physical activity and sedentary time

Accelerometery has become a common objective tool in contemporary research focusing on physical activity. In the current study, physical activity was assessed using an Actigraph wGT3X-BT monitor accelerometer (Actigraph, Pensacola, FL, USA). It is a small electronic device designed to record acceleration associated with body movement. The participants wore the accelerometer for seven consecutive days. The device was placed on the participant’s right hip. Participants were informed to remove the accelerometer only for sleep at night, and for bathing or swimming. The acquired data was computed using the dedicated Actilife software (Actilife software, version 6.8.3; ActiGraph). The older adult specific cut-points were used as follows: sedentary (< 50 counts/minute), moderate-to-vigorous physical activity (MVPA ≥ 1041 counts/minute)^13,14^.

For the purposes of the analyses, participants were divided into two groups: (1) those who met WHO recommendations regarding the level of PA and (2) those who did not^15^.

### Socio-demographic/lifestyle factors

Socio-economic and lifestyle factors (age, date of birth, place of residence, occupation, education, and smoking) were investigated using a self-report technique. Due to the fact that the study group comprised individuals who were entitled to retire from work, with regard to their occupational status the participants were divided into two categories, i.e., occupationally active and retired. The participants’ educational level was classified to correspond with three categories, making reference to International Standard Classification of Education (ISCED 2011): primary (equivalent to ISCED 1 and ISCED 2), secondary (ISCED 3, ISCED 4, ISCED 5) and higher (ISCED 6, ISCED 7, ISCED 8)^16,17^.

### Statistical analysis

Statistical analysis was performed using the R Statistical Software (version 4.1.1.; R Foundation for Statistical Computing, Vienna, Austria)^18^. The statistics are presented as median values (Me), and quartiles (Q) for quantitative variables, and n (%) for categorical variables. The normality of the distributions was tested using the Kolmogorov–Smirnov test with Lilliefors correction. In the case of all the variables analysed, their distributions significantly differed from the normal distributions. Chi-squared test (with Yates’ correction for 2 × 2 tables) was applied to compare the qualitative variables in the groups. If the values in contingency tables were low, Fisher’s exact test was used instead. The comparison of the values of quantitative variables in three or more groups was performed using the Kruskal–Wallis test. The Mann–Whitney test was applied to compare quantitative variables between two groups. Correlations between quantitative variables were analysed using the Spearman correlation coefficient. Logistic regression analyses were also performed using the input method and the stepwise method. The regression was performed separately for women and men, and separately for age groups. The factors analysed included: the dependent variable of hypertension (n = 47); quantitative independent variables of average MVPA, sedentary time, BFP, and BMI; as well as the categorical independent variable of Compliance with PA recommendations (1-no). The level of statistical significance was adopted at p < 0.05.

## Results

The final sample consisted of 109 participants aged from 60 to 85 years (mean age 67.3 years; 70.4% females). There were no significant difference in sex, age, occupation and education between participants from urban vs. rural area (Table 1).

**Table 1:** Characteristics of the study population.

Parameter		Place of residence			*p*
		Urban, n (%)	Rural, n (%)	Total, n (%)	
Sex	Female	38 (70.4)	42 (76.4)	80 (73.4)	0.623
	Male	16 (29.6)	13 (23.6)	29 (26.6)	
Age	60–64 years	20 (37.0)	23 (41.8)	43 (39.5)	0.610
	65–85 years	34 (63.0)	32 (58.2)	66 (60.6)	
Occupation	Retired	48 (88.9)	50 (90.9)	98 (89.9)	0.974
	Occupationally active	6 (11.1)	5 (9.1)	11 (10.1)	
Education	Primary	5 (9.3)	9 (16.4)	14 (12.8)	0.436
	Secondary	39 (72.2)	34 (61.8)	73 (67.0)	
	Higher	10 (18.6)	12 (21.8)	22 (20.12)	

Pearson chi-square test has been used.

Table 2 shows in what way BMI, BFP, sedentary time and average MVPA are related to blood pressure values. BMI correlates significantly and positively with average SBP and DBP (higher BMI corresponds to higher blood pressure). The same associations were found in the case of BFP, i.e., higher BFP values correspond to higher blood pressure values, both SBP and DBP. The above associations were observed in both women and men (Table 2).

**Table 2:** Associations between blood pressure values and BMI, BFP, sedentary time as well as MVPA.

Parameter	Spearman's correlation coefficient					
	Total		Female		Male	
	r	p	r	p	r	p
BMI						
SBP (mm Hg)	0.567	** < 0.001**	0.450	** < 0.001**	0.758	** < 0.001**
DBP (mm Hg)	0.556	** < 0.001**	0.502	** < 0.001**	0.598	**0.001**
BFP						
SBP (mm Hg)	0.379	** < 0.001**	0.227	**0.043**	0.693	** < 0.001**
DBP (mm Hg)	0.378	** < 0.001**	0.294	**0.008**	0.590	**0.001**
Sedentary time						
SBP (mm Hg)	0.109	0.257	0.001	0.995	0.317	0.094
DBP (mm Hg)	0.018	0.851	-0.030	0.789	0.136	0.482
Average MVPA per day						
SBP (mm Hg)	0.130	0.179	0.125	0.269	0.108	0.577
DBP (mm Hg)	0.038	0.697	0.033	0.773	0.041	0.834

Significant associations are highlighted in bold.

*BMI* body mass index, *BFP* body fat percentage, *DBP* diastolic blood pressure, *MVPA* moderate to vigorous physical activity, *SBP* systolic blood pressure.

Table 3 shows associations between the category of blood pressure and BMI, BFP category as well as the level of physical activity. The highest median SBP and DBP were found in participants with obesity, and the lowest in those with normal body weight assessed based on BMI values. Similarly, the median SBP and DBP values were higher in participants with excess adiposity and lower in those with normal adiposity, as assessed by BFP. Moreover, hypertension was more frequently observed in participants with obesity and excess adiposity (84.6% and 54.7%, respectively). None of the participants with obesity had optimal blood pressure (Table 3). In the table Suppl. S1 are presented the relationships between blood pressure category and BMI, BFP as well as the level of physical activity divided by age and gender.

**Table 3:** Associations between the type of blood pressure and BMI category, BFP category as well as the level of physical activity.

Parameter		SBP (mm Hg)		DBP (mm Hg)		Blood pressure category			
		Me	Q	Me	Q	Optimal, n (%)	Normal, n (%)	High normal, n (%)	Hypertension, n (%)
BMI category	Normal	120	117–123	81	78–84	9 (36.0)	10 (40.0)	2 (8.0)	4 (16.0)
	Overweight	137	124–139	88	85–89	3 (6.7)	7 (15.6)	25 (55.6)	10 (22.2)
	Obesity	168	150.5–185	99	93 – 104.5	0 (0.0)	1 (2.6)	5 (12.8)	33 (84.6)
*p*		** < 0.001**		** < 0.001**		** < 0.001**			
BFP category	No excess adiposity	124	120–139	86	82–89	8 (17.8)	11 (24.4)	14 (31.1)	12 (26.7)
	Excess adiposity	143.5	132–178	93	86–99.25	4 (6.3)	7 (11.0)	18 (28.1)	35 (54.7)
*p*		** < 0.001**		**0.004**		**0.011**			
Compliance with PA recommendations	Yes	136	123–163.25	89	84–97	7 (9.7)	13 (18.1)	26 (36.1)	26 (36.1)
	No	153	122–178.5	93	82.5–104.5	5 (13.5)	5 (13.5)	6 (16.2)	21 (56.8)
*p*		0.189		0.154		0.096			

The analyses were performed using Mann–Whitney U test (for BPF and compliance with PA recommendations), the Kruskal–Wallis test (for BMI), Pearson chi-square (for blood pressure).

*BMI* body mass index, *BFP* body fat percentage, *DBP* diastolic blood pressure, *Me* median, *PA* physical activity, *SBP* systolic blood pressure, *Q* quartiles.

Significant associations are highlighted in bold.

The analysis of the risk factors for hypertension, in Table 4, has found significant associations only in the case of the level of education. Hypertension was most common in participants with secondary education, and the problem was least frequent in those with primary education (p = 0.017) (Table 4).

**Table 4:** Associations between hypertension and sociodemographic factors.

Parameter	Group	Blood pressure category				*p*
		Optimal, n (%)	Normal, n (%)	High normal, n (%)	Hypertension, n (%)	
Education	Primary (n = 14)	2 (14.3)	2 (14.3)	7 (50.0)	3 (21.4)	**0.017**
	Secondary (n = 73)	4 (5.45)	15 (20.56)	18 (24.7)	36 (49.3)	
	Higher (n = 22)	6 (27.2)	1 (4.56)	7 (31.8)	8 (36.4)	
Occupational status	Retired (n = 98)	12 (12.2)	16 (16.3)	27 (27.6)	43 (43.9)	0.511
	Occupationally active (n = 11)	0 (0.0)	2 (18.2)	5 (45.5)	4 (36.4)	
Age	Up to 64 years (n = 43)	6 (14.0)	8 (18.6)	13 (30.2)	16 (37.2)	0.719
	65–85 years (n = 66)	6 (9.1)	10 (15.2)	19 (28.8)	31 (47.0)	
Sex	Female (n = 80)	7 (8.8)	12 (15.0)	25 (31.3)	36 (45.0)	0.482
	Male (n = 29)	5 (17.2)	6 (20.7)	7 (24.1)	11 (37.9)	
Place of residence	Urban (n = 54)	3 (5.6)	13 (24.1)	15 (27.8)	23 (42.6)	0.082
	Rural (n = 55)	9 (16.4)	5 (9.1)	17 (30.9)	24 (43.6)	

Significant associations are highlighted in bold; Pearson chi-square test has been used.

**Figure 1:**
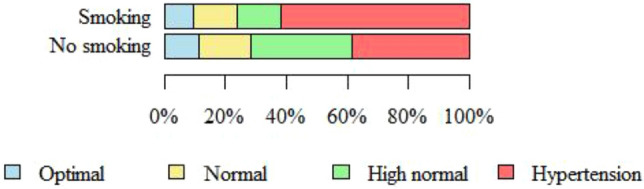
Blood pressure category in relation to smoking.

Figure 1 shows blood pressure categories in relation to smoking. More than half (61.9%) of the smoking participants had hypertension, however the relationship between smoking and blood pressure category was not statistically significant (p = 0.238) (Fig. 1).

A logistic regression analysis was performed to check which of the investigated factors had the strongest association with hypertension identified in women and men, and in different age groups. The model of the input method showed that the risk of hypertension was 10 times higher in women who did not meet the PA recommendations. Higher BMI was associated with a greater risk of hypertension in women, and in all participants aged 60–64 years. In the group aged 65–85 years, a greater risk for hypertension was associated with a lack of compliance with PA recommendations (OR = 5.10), and with higher BMI (OR = 1.47); in this group the risk was also slightly higher (by 2%) in participants with higher MVPA. However, according to stepwise regression, MVPA was non-significant. The stepwise method showed that hypertension was over seven times higher in women who did not meet the PA recommendations. In men, the strongest association was found between hypertension and BFP (OR = 1.30). Furthermore, higher BMI was the strongest risk factor for hypertension in both age groups (OR = 1.30 for 60–64 years, OR = 1.37 for 65–85 years) (Table 5).

**Table 5:** Factors associated with hypertension, in logistic regression analysis based on input method and stepwise method.

Groups	Variables	B	SE	Wald	df	p	OR
Multiple regression (multiple input variables)							
Female	MVPA per day [min]	0.019	0.011	3.180	1	0.075	1.02 (1.00–1.04)
	Sedentary time [min]	0.000	0.000	0.926	1	0.336	1.00 (1.00–1.00)
	Compliance with PA recommendations (no)	2.307	0.786	8.620	1	**0.003**	10.05 (2.15–46.89)
	BFP [%]	− 0.122	0.075	2.641	1	0.104	0.88 (0.76–1.03)
	BMI [kg/m^2^]	0.457	0.126	13.244	1	**0.000**	1.58 (1.23–2.02)
Male	MVPA per day [min]	− 0.083	0.053	2.456	1	0.117	0.92 (0.83–1.02)
	Sedentary time [min]	− 0.002	0.001	2.700	1	0.100	1.00 (1.00–1.00)
	Compliance with PA recommendations (No)	− 6.333	3.691	2.944	1	0.086	0.00 (0.00–2.46)
	BFP [%]	0.401	0.261	2.362	1	0.124	1.49 (0.90–2.49)
	BMI [kg/m^2^]	0.314	0.281	1.246	1	0.264	1.37 (0.79–2.37)
60–64 years	MVPA per day [min]	− 0.005	0.013	0.156	1	0.693	0.99 (0.97–1.02)
	Sedentary time [min]	0.000	0.000	0.708	1	0.400	1.00 (1.00–1.00)
	Compliance with PA recommendations (No)	1.259	0.923	1.861	1	0.172	3.25 (0.58–21.49)
	BFP [%]	− 0.031	0.067	0.209	1	0.648	0.97 (0.85–1.11)
	BMI [kg/m^2^]	0.341	0.146	5.461	1	**0.019**	1.41 (1.06–1.87)
65–85 years	MVPA per day [min]	0.023	0.012	3.953	1	**0.047**	1.02 (1.00–1.05)
	Sedentary time [min]	0.000	0.000	0.103	1	0.748	1.00 (1.00–1.00)
	Compliance with PA recommendations (no)	1.629	0.818	3.966	1	**0.046**	5.10 (1.03–25.32)
	BFP [%]	− 0.013	0.052	0.061	1	0.805	0.99 (0.89–1.09)
	BMI [kg/m^2^]	0.386	0.114	11.504	1	**0.001**	1.47 (1.18–1.84)
Stepwise regression*							
Female	Compliance with PA recommendations (No)	2.046	0.697	8.611	1	**0.003**	7.73 (1.97–30.32)
	BMI [kg/m^2^]	0.299	0.076	15.440	1	**0.000**	1.35 (1.16–1.56)
Male	BFP [%]	0.263	0.102	6.674	1	**0.010**	1.30 (1.07–1.59)
60–64 years	BMI [kg/m^2^]	0.263	0.096	7.553	1	**0.006**	1.30 (1.08–1.57)
65–85 years	BMI [kg/m^2^]	0.314	0.086	13.287	1	**0.000**	1.37 (1.16–1.62)

Significant associations are highlighted in bold.

*BMI* body mass index, *BFP* body fat percentage, *PA* physical activity.

*The final model was obtained after two steps.

## Discussion

Arterial hypertension has consistently, for many years, been one of the leading and most common causes of heart attack, stroke, and kidney failure significantly increasing the risk of premature mortality. Hypertension is recognised in medical literature as a significant and complex health problem, but it should be remembered that it is a modifiable condition that can only be ameliorated by applying well-designed combination treatment and implementing appropriate healthy habits. Despite the numerous advances in medicine related to the treatment of the condition, the European Society of Hypertension (ESH) confirms that arterial hypertension is a concern for as many as 1.28 billion adults between 30 and 79 years worldwide. The problem is more and more widespread, whereas the significance of the most common risk factors often differs from one place to another across the world. Two-thirds of those affected by hypertension live in low-income and middle-income countries^9^. The WHO confirmed that in 2019, compared to the global average, Poland had a higher hypertension prevalence in adults aged 30–79 years, with the estimated 49% and 56% rates in females and males, respectively^19^. In view of the above, the authors of the current study decided to assess the current prevalence of hypertension, in the context of various risk factors, which they investigated in a group of adults from south–eastern Poland.

The present study provides up-to-date evidence reflecting the large scale of the problem of hypertension among people mainly over 65 years of age who have not been diagnosed with other serious health conditions. Elderly people are particularly notable because of the very high prevalence of hypertension observed in this group, contributing to increasing morbidity and mortality rates in this population. Important age threshold is at 65 years when many people retire from work. It is estimated that acceleration of arterial aging is observed at that age, leading to an increase in SBP, a decrease in DBP, and consequently an increase in pulse pressure^20^. Furthermore, majority of these patients have no symptoms, due to which it is necessary to regularly screen not only those with other health conditions but all individuals over 65 years of age. In the whole sample examined in this study, hypertension was observed at a mean rate of 40%, however the value increased significantly with the BMI category, i.e., from 16% in the individuals with normal body weight to 22% in those with overweight and 85% in individuals with obesity, which is consistent with other research reports from our country. According to Polish official epidemiological research conducted during the last two decades, the problem is observed in 29% to 45% of the adult populations, however the prevalence of hypertension significantly increases with age, and those over 65 years of age may be affected even at a high rate of 75%. The data from the latest ESH report (2023), summarizing the existing evidence, confirm that in 2019, the global age-standardised average prevalence of hypertension in adults below 79 years of age amounted to 34% in men and 32% in women. The current data are related to adults representing the oldest age group, and were acquired in the Podkarpackie Region where very large increase in the prevalence of this medical condition is predicted by 2029. According to the latest report published by the National Health Fund in 2019, the Podkarpackie Region had one of the highest prevalence rates, i.e., 307 cases per 1,000 population, compared to the highest rate of 397 cases found in the Lubuskie Region; these numbers reflect the severity of the problem existing throughout Poland^21^. However, the last comprehensive study investigating a large Polish population dates from 2017, and since that time the findings have not been updated taking into account the specific regions of Poland in order to illustrate the scale of the problem; this explains the subject matter of this study. Long-term worldwide observations conducted during 1990–2019 show that Poland ranks at number five in the world as regards the significance of increase in the prevalence of hypertension (35%)^22^.

Arterial hypertension depends on the multifactorial and complex interaction between both genetic background, aging and environmental factors. Maintaining a healthy lifestyle, mainly involving age-appropriate physical activity, good eating habits, weight control and stress management are key to preventing or delaying the onset of hypertension and its dangerous consequences. Individuals with good health-related habits were found to have a 4–5 mmHg lower BP, irrespective of any BP genetic risk, compared to their peers with unfavourable lifestyles^23^. The most recent risk factor taken into account in the data analyses is air and noise pollution in urbanised societies, which is indicative of a changing world imposing increasingly altered living conditions that are not always beneficial to health. A new element of the discussions is related to the need to preserve the environment and pay attention to ecology which provide a foundation for people to stay healthy^24^.

The present findings show that individuals’ body weight and age continue to be among the key factors associated with hypertension. It has been established that obesity contributes to an increase in low-density lipoprotein (LDL), a decrease in high-density lipoprotein (HDL), and to lower glucose tolerance as well as higher insulin resistance, all of these factors promoting development of hypertension. The current study demonstrates that values of both SBP and DBP are significantly related to BMI and BFP (%). The results clearly show the highest mean SBP and DBP values in the group of individuals with obesity, and the lowest in those with normal body weight reflected by BMI values. None of the participants with obesity had ideal blood pressure. Similar observations were reported by Shukuri et al. who estimated that overweight and obese older adults were even 4.29 times more likely to be hypertensive compared to their normal-weight counterparts^25,26^. The prevalence of hypertension and its association with BMI, Triglycerides (TG) level, and other factors in individuals aged ≥ 60 years were investigated by Zhang et al., who also reported that overweight and obese men and women were more likely to be affected by hypertension. What is more, individuals aged ≥ 68 years exhibited lower effects of BMI level on BP^27^. Similar conclusions were presented by Li et al. who found that the association between blood pressure and body composition indices was weaker in the elderly compared to the younger subjects^28^.

The study also investigated other selected and important risk factors for hypertension, i.e., education, occupation, age, sex, place of residence, smoking, MVPA and sedentary time. It was found that education level was the strongest risk factor. Hypertension was most common in participants with secondary education, and least frequent in those with primary education. The results suggest that those with lower education were less at risk of hypertension. The authors believe this may be linked to a different lifestyle, and the associated level of perceived stress. Perhaps individuals with higher education take up jobs associated with higher positions and greater stress, which might explain higher rate of hypertension in this group. Likewise, a study by Chia et al. showed that individuals with primary or lower education more effectively complied with BP treatment process^29^. However, conflicting evidence has been reported in the literature. For instance, according to Uchmanowiczet al., individuals with higher education were more likely to adhere to the medication regime, while those with no education or primary, or secondary education presented lower adherence rates with regard to routine medical appointments^30^. Likewise, in the study by Di Chiara et al., education was independently associated with global cardiovascular risk^31^. It would be necessary to cross-reference the findings acquired to the prevalence of this condition, as perhaps this health problem was more common in well-educated people. Van Oort et al. emphasise the possitive effect of higher education level on hypertension risk because of more resources available to maintain a healthy lifestyle^32^. Regardless of the differences in the results acquired, it is important to raise awareness of the problem in the general public, irrespective of their education, about the ways to prevent hypertension. Every professional working with this group of patients should remember it is necessary to consistently educate the patients and inform them abour the possible negative health consequences, and finally to establish interventions for improving patient outcomes^33^.

The present study has not confirmed the strength of factors connected with smoking and sedentary lifestyle. By comparison, Lu et al. did not find differences in sex, marital status, education, and income between subjects in normotensive and hypertensive groups. The factors presenting the highest risk of hypertension are associated with family history of hypertension and higher BMI^34^. Analysis of sedentary time in the study group found no associations with SBP or DBP. Agarwal et al. reported that 58% of the geriatric population in their study were sedentary, which supports the claim that physical inactivity is a notable risk factor for increased BP^35^. The most recent study by Sri Hari et al., focusing on risk factors for hypertension in elderly people, showed the strongest relationship of the condition with family history of hypertension, as well as physical inactivity and obesity, waist circumference, and waist hip ratio^36^. Although the current study has not shown significant associations with other risk factors, apart from BMI and FBP, their role in development of hypertension should not be underestimated. However, they require more in-depth investigation in larger populations, and they all should be taken into account in measures intended to prevent hypertension in older people.

Limitations of the study relate to the fact that it does not present data informing about the participants’ diet, amount of salt consumed, physical activity, mental well-being, level of stress experienced, as well as lipid abnormalities. Understanding the risk factors for hypertension, and the effects of BMI and age on BP values may be particularly important for preventing mortality due to cardiovascular diseases^37^. Hypertension is a potentially modifiable medical problem, therefore it is necessary to control its prevalence and to introduce educational methods and preventive measures particularly among those most at risk, i.e., individuals aged 65 or more^38^. Another limitation of the study may be linked to the use of BMI in older population. Some experts argue that current BMI thresholds for overweight/obesity may be overly restrictive in the case of elderly people, and they claim that BMI is of limited usefulness in the elderly population because this measure does not allow us to distinguish between a lean body mass and a fat mass^39^. In view of this, the more objective measure of BFP has also been applied in the study. A limitation of this study is also the small number of study group (109 participants). Despite of characteristics that ensure G-power in the research, there should be the stronger representation of men (in comparison with women) and better comparative representation by age groups and their education. The study also did not include collecting information about taking medications of the participants, so the results would be different if the study group was divided into two subgroups (taking and not taking blood pressure drugs).

The present article confirms the high prevalence of hypertension in adult Poles living in the Podkarpackie Region and emphasises the role of controlling body weight by implementing appropriate healthy habits. Notably, however, lifestyle changes should never delay the antihypertensive drug therapy to optimize the related benefits of BP reductions. All these measures, including pharmacotherapy and adherence to healthy lifestyle are indispensable in combating the more and more common arterial hypertension.

## Conclusions

The highest prevalence of hypertension is significantly related to obesity and excess adiposity. The level of education was a strong risk factor for hypertension in the study group. Logistic regression analyses showed that hypertension was more common in participants with a higher BMI, and in those who did not meet PA recommendation.

### Supplementary Information

Supplementary Table 1.

## Data Availability

The datasets generated and/or analysed during the current study are not publicly available due to the requirement to protect the participants’ privacy, but are available from the corresponding author on reasonable request.
