# Supplementary material for: Risk factors and prevalence of hypertension in older adults from south-eastern Poland: an observational study
Source: Sci Rep. 2024 Jan 16;14:1450. doi: 10.1038/s41598-024-52009-3 (PMC10792009; doi:10.1038/s41598-024-52009-3)
Supplement: Supplementary file 1 — Supplementary Table 1. [file 41598_2024_52009_MOESM1_ESM.docx]

Suppl. 1. Associations between the type of blood pressure and BMI category, BFP category as well as the level of physical activity divided by age and gender.

| **Parameter** | | **Female** | | | | | **Male** | | | |
| --- | --- | --- | --- | --- | --- | --- | --- | --- | --- | --- |
|  |  | **Blood pressure category** | | | | | **Blood pressure category** | | | |
|  |  | **Optimal**  **n (%)** | **Normal**  **n (%)** | **High normal**  **n (%)** | | **Hypertension**  **n (%)** | **Optimal**  **n (%)** | **Normal**  **n (%)** | **High normal**  **n (%)** | **Hypertension**  **n (%)** |
| BMI category | Normal | 5 (33.3) | 6 (40.0) | 1 (6.7) | | 3 (20.0) | 4 (40) | 4 (40) | 1 (10.0) | 1 (10.0) |
|  | Overweight | 2 (6.1) | 5 (15.2) | 19 (57.6) | | 7 (21.2) | 1 (8.3) | 2 (16.7) | 6 (50.0) | 3 (25.0) |
|  | Obesity | 0 (0.0) | 1 (3.1) | 5 (15.6) | | 26 (81.3) | 0 (0.0) | 0 (0.0) | 0 (0.0) | 7 (100.0) |
| *p* | | **<0.001** | | | | | **0.001** | | | |
| BFP category | No excess adiposity | 3 (13.6) | 5 (22.7) | 7 (31.8) | 7 (31.8) | | 5 (21.7) | 6 (26.1) | 7 (30.4) | 5 (21.7) |
|  | Excess adiposity | 4 (6.9) | 7 (12.1) | 18 (31.0) | 29 (50.0) | | 0 (0.0) | 0 (0.0) | 0 (0.0) | 6 (100.0) |
| *p* | | 0.360 | | | | | **0.006** | | | |
| Compliance with PA recommendations | Yes | 5 (8.6) | 11 (19.0) | 22 (37.9) | | 20 (34.5) | 2 (14.3) | 2 (14.3) | 4 (28.6) | 6 (42.9) |
|  | No | 2 (9.1) | 1 (4.5) | 3 (13.6) | | 16 (72,7) | 3 (20.0) | 4 (26.7) | 3 (20.0) | 5 (33.3) |
| *p* | | 0.015 | | | | | 0.785 | | | |

**Age**

| **Parameter** | | **60-64 years** | | | | | | **65-85 years** | | | |
| --- | --- | --- | --- | --- | --- | --- | --- | --- | --- | --- | --- |
|  |  | **Blood pressure category** | | | | | | **Blood pressure category** | | | |
|  |  | **Optimal**  **n (%)** | **Normal**  **n (%)** | | **High normal**  **n (%)** | | **Hypertension**  **n (%)** | **Optimal**  **n (%)** | **Normal**  **n (%)** | **High normal**  **n (%)** | **Hypertension**  **n (%)** |
| BMI category | Normal | 5 (33.3) | 5 (33.3) | | 2 (13.3) | | 3 (20.0) | 4 (40.0) | 5 (50.0) | 0 (0.0) | 1 (10.0) |
|  | Overweight | 1 (6.3) | 2 (12.5) | | 10 (62.5) | | 3 (18.8) | 2 (6.9) | 5 (17.2) | 15 (51.7) | 7 (24.1) |
|  | Obesity | 0 (0.0) | 1 (8.3) | | 1 (8.3) | | 10 (83.3) | 0 (0.0) | 0 (0.0) | 4 (14.8) | 23 (85.2) |
| *p* | | **<0.001** | | | | | | **<0.001** | | | |
| BFP category | No excess adiposity | 4 (18.2) | | 6 (27.3) | 7 (31.8) | 5 (22.7) | | 4 (17.4) | 5 (21.7) | 7 (30.4) | 7 (30.4) |
|  | Excess adiposity | 2 (9.5) | | 2 (9.5) | 6 (28.6) | 11 (52.4) | | 2 (4.7) | 5 (11.6) | 12 (27.9) | 24 (55.8) |
| *P* | | 0.174 | | | | | | 0.123 | | | |
| Compliance with PA recommendations | Yes | 5 (15.6) | | 6 (18.8) | 11 (34.4) | | 10 (31.3) | 2 (5.0) | 7 (17.5) | 15 (37.5) | 16 (40.0) |
|  | No | 1 (9.1) | | 2 (18.2) | 2 (18.2) | | 6 (54.5) | 4 (15.4) | 3 (11.5) | 4 (15.4) | 15 (57.7) |
| *p* | | 0.541 | | | | | | 0.113 | | | |

BMI – body mass index, BFP – body fat percentage, PA – physical activity; significant associations are highlighted in bold; The analyses were performed using Pearson chi-square test
